# In search of a Goldilocks zone for credible AI

**DOI:** 10.1038/s41598-021-93109-8

**Published:** 2021-07-01

**Authors:** Kevin Allan, Nir Oren, Jacqui Hutchison, Douglas Martin

**Affiliations:** 1 School of Psychology, University of Aberdeen, Aberdeen, AB24 2UB UK; 2 School of Natural and Computing Sciences, University of Aberdeen, Aberdeen, AB24 2UB UK

**Keywords:** Psychology, Human behaviour

## Abstract

If artificial intelligence (AI) is to help solve individual, societal and global problems, humans should neither underestimate nor overestimate its trustworthiness. Situated in-between these two extremes is an ideal ‘Goldilocks’ zone of credibility. But what will keep trust in this zone? We hypothesise that this role ultimately falls to the social cognition mechanisms which adaptively regulate conformity between humans. This novel hypothesis predicts that human-like functional biases in conformity should occur during interactions with AI. We examined multiple tests of this prediction using a collaborative remembering paradigm, where participants viewed household scenes for 30 s vs. 2 min, then saw 2-alternative forced-choice decisions about scene content originating either from AI- or human-sources. We manipulated the credibility of different sources (Experiment 1) and, from a single source, the estimated-likelihood (Experiment 2) and objective accuracy (Experiment 3) of specific decisions. As predicted, each manipulation produced functional biases for AI-sources mirroring those found for human-sources. Participants conformed more to higher credibility sources, and higher-likelihood or more objectively accurate decisions, becoming increasingly sensitive to source accuracy when their own capability was reduced. These findings support the hypothesised role of social cognition in regulating AI’s influence, raising important implications and new directions for research on human–AI interaction.

## Introduction

Across the planet huge efforts are underway to build artificial intelligences (AI) capable of matching or surpassing human thought. When well-intentioned, the common goal is to produce credible AI that generate accurate decisions, using various symbolic or, increasingly, machine-learning based methods. Rolled out across society, the anticipated impact of AI is immense and varied^1,2^ but co-exists with evidence of mixed public perceptions^3^ as well as doubts about institutional governance^4^. In response, there are now many systematic reviews, particularly in medicine, that cover AI’s history and current capabilities^5–8^, its actual performance upon specific problems^9,10^ and various ethical and legal frameworks that may evolve^11–13^. Arising from these reviews is the awareness of a critical need for research on how AI influences human decision-making, and consequently how that influence can be controlled to obtain the anticipated benefits without incurring the perceived costs.

For systems intended to improve rather than degrade or confuse human knowledge, the anticipated benefit is predicated on being appropriately persuasive, which we will refer to as operating within a ‘Goldilocks’ zone of credibility. In the Goldilocks zone, the persuasive influence of AI does not stray beyond its capability, generating an appropriate level of trust that, for essentially all forms of non-human agent, is highly desirable but difficult to implement^14,15^. In essence, though, decisions or advice likely to be less accurate should be less persuasive and vice versa. This means that an AI would seek to persuade on the basis of its actual or presumed reputation for accuracy, or its’ own estimated likelihood of being correct. Subject solely to these factors, a well-engineered system would operate in the Goldilocks zone, and conformity to the knowledge it provided would be just right, neither too much nor too little. But this entirely begs the question of what will happen when a person can freely choose whether to sacrifice their autonomy in order to gain the consistency and accuracy offered by AI.

AI themselves can of course operate with different degrees of autonomy, and to begin with it is useful to briefly consider the types of system of concern to us here. Our focus is on systems operating with partial or conditional autonomy to provide intelligent judgements that support human behaviour and decision-making, of which there are many different kinds^2^. Some are embodied, e.g. in vehicles or drones, and perform highly complex tasks involving multiple faculties analogous to human perception, attention and memory, that culminate directly in action (e.g. automated driving) or recommendations for action (e.g. to return to human control). Whereas, other semi or partially autonomous applications mimic specific human faculties, providing particular forms of knowledge or insight that, prior to modern developments in AI, were only available via interactions with other people. At present, perhaps the most societally important of these involve diagnostic classification of medical images by AI^6–9^, and the use of facial recognition AI for forensic or surveillance purposes^16^. Underwriting these applications is the use of image recognition and classification to support inferences about depicted persons, objects or events, with varying degrees of accuracy and bias. In such cases, how will humans decide when to conform to an AI’s judgement?

Humans face a similar choice, whether to conform or not, whenever we encounter one another’s knowledge or opinions. In such cases, psychologists have long-established^17^ evidence that conformity is not a random act, but is instead biased by two broad types of social influence^18^ with deep biological roots^19–23^. ‘Informational’ influence^18,24–26^ rests upon an individual’s need to maintain an accurate mental model of reality, triggering conformity to other individuals’ knowledge of how the world is or was or will be. In contrast, ‘normative’ influence^18,27–29^ rests upon the need to seek agreement with others, triggering conformity that signals one’s allegiance to social norms. The internal, cognitive, regulation of these social influences has recently been intensively studied to address forensic concerns about the malleability of witness memory under social pressure^30,31^. Various factors promoting or inhibiting conformity to other peoples’ knowledge have consequently been examined, usually from the theoretical perspective that social influences cause malfunctions that corrupt and distort one’s knowledge^32–36^.

From a wider biological perspective, however, both types of social influence take on a more functional character^19,20,24,37–39^. Indirect benefits for social cohesion and cooperation between individuals may arise from normative influence, while direct benefits from an improved mental model of reality may arise from informational influence. Focussing on informational influence, it has been consistently reported that conformity is functionally biased towards individuals likely to provide knowledge complimentary or superior to our own. Conformity increases have been demonstrated as the likely accuracy or credibility of one’s partner increases, and vice versa^25,40–42^. While conformity to others is increased when the accuracy of one’s own memory decreases, and vice versa^43–45^. Based on these results, we^24,37^ and others^41,46^ proposed that memory conformity is in fact adaptively biased by explicit mentalizing mechanisms^19^ that provide insight into the knowledge, beliefs, and intentions of others relative to one’s own.

To test this proposal^24^ we asked young healthy adult participants to interact via computer on a collaborative test of memory for household scenes. One group of participants was told that their partner always had twice as much time to view each image, and another group of participants was told that their partner always had only half as much time. Each participant was shown accurate and errant simulated partner decisions about image content on some trials before giving their own decision. Baseline memory performance, when no partner response was available, showed that restricting participants own viewing time impaired their memory, accompanied by increased conformity to partner’s responses. But, critically, this only occurred in the group informed that their partner had viewed the images for twice as long as they did themselves. In the other group, informed that their partner had half as long to view images, declines in baseline memory were not accompanied by increased reliance on their partners potentially even worse memory. These results provided the first demonstration that informational influence is regulated so that it remains commensurate with the likely accuracy of the source, relative to one’s current epistemic need. This, in turn, provides a way to operationalise the notion of a Goldilocks zone of credibility.

The primary aim of the present three experiments is to establish whether conformity to knowledge offered by AI is driven by the same regulatory mechanisms, thereby maintaining its influence within a Goldilocks zone of credibility. In experiment 1, we begin by examining whether conformity to AI within the memory conformity paradigm follows the ‘classic’ pattern reviewed above, i.e. sensitivity to one’s own credibility and that of the information source. Following the 30 s vs. 2 min scene viewing manipulation^24^, questions about scene content were provided to young, healthy adult participants, with one group informed that they will receive help from an image-classifying AI that has ‘viewed’ the scenes, and another control group informed that help comes from a human partner who has viewed the scenes. This allows us to directly compare and contrast the effect of partner type between groups.

The credibility of the AI and human control is then manipulated immediately prior to the memory test, by providing information about their reputation for accuracy. Using a counterbalanced order across participants, in one block image viewing is followed by a memory test in collaboration with AI/partner described as accurate in six out of ten cases, and in another block with AI/partner described as accurate in eight out of ten cases. Finally, after each memory test, participants also rate how reliable their own memory was compared to that of their partner, allowing us to quantify the subjective impact of the experimental manipulations on trust in each source.

As detailed below, in experiment 1 a key finding is that reputation does indeed alter the influence of the AI and human partners in identical ways, however reducing participants baseline accuracy does not increase reliance upon AI/human partners with a higher vs. lower reputation for accuracy. This indicates that reputational signals alone are not sufficient to enhance trust when epistemic need is increased. Experiments 2 and 3 then go on to show that increased epistemic need can lead to enhanced trust in AI (and human) agents, but this requires the AI (and human) to communicate at a per-decision level—by introducing per-decision likelihoods in experiment 2, and by increasing the objective accuracy of the per-decision likelihood information in experiment 3. Most critically, identical patterns of influence are consistently observed for AI and human partners. This strongly suggests that the informational influence of both types of agent is regulated by similar cognitive mechanisms, which can be constrained to operate within a Goldilocks zone commensurate to the accuracy of the source and the epistemic need of the receiver.

## Experiment 1: source credibility effects

### Results

**Figure 1:**
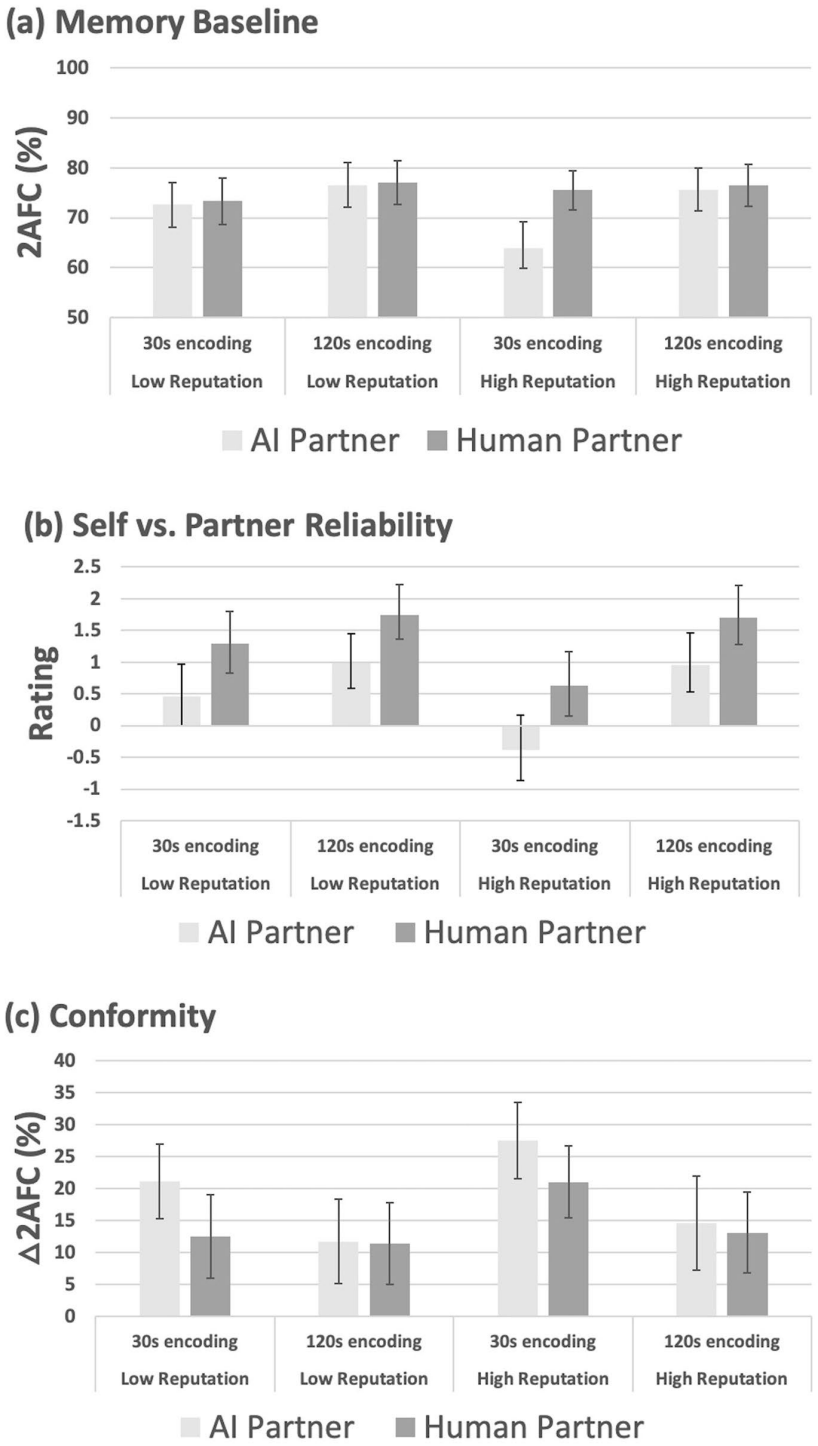
Data for Experiment 1 (Source credibility effects). (**a**) Mean percentage baseline memory 2-alternative forced choice (2AFC) performance (± 95% CI), in the AI (light-grey) vs. human (dark-grey) groups following 30 s or 120 s viewing time at encoding, for low (six out of ten correct) vs. high reputation (eight out of ten correct) AI or human partner. (**b**) Mean perceived reliability ratings (± 95% CI) for self vs. AI or human partner. Positive values reflect relative reliability of self, negative values reflect relative reliability of AI or human partner (zero indicates equivalent perceived reliability). (**c**) Change in mean percentage 2AFC (**△**2AFC, ± 95% CI) performance induced by conformity to accurate minus errant information following 30 s vs. 120 s viewing time at encoding, for AI vs. human partners of low vs. high reputation.

2-Alternative forced-choice (2AFC) baseline accuracy (Fig. 1a) was significantly reduced by 30 s vs. 2 min viewing time at encoding (F(1, 101) = 12.73, p = 0.001, ηp^2^ = 0.115). This effect was insensitive to the between-subjects factor of AI vs. human-partner (F(1, 98) = 1.79, p = 0.184, ηp^2^ = 0.018). Source reputation also altered baseline memory, which was significantly lower in tests performed with the reputedly less accurate source (F(1, 98) = 4.08, p < 0.046, ηp^2^ = 0.04).

Across all conditions, when receiving help from AI the participants rated their own memory as relatively less reliable, compared to when human help was received (Fig. 1b, F(1, 98) = 16.95, p = 0.000096, ηp^2^ = 0.144). The subjective reliability data also revealed main effects of source reputation (F(1, 98) = 4.92, p = 0.029, ηp^2^ = 0.048) and encoding time (F(1, 98) = 31.14, p < 0.00001, ηp^2^ = 0.241) that interacted (F(1, 98) = 6.05, p = 0.016, ηp^2^ = 0.058). With images viewed for only 30 s, participants rated their own memory as relatively less reliable than that of the reputedly more vs. less accurate source (t(99) = 3.10, p = 0.003), but source reputation did not influence subjective ratings of memory reliability with images viewed for 2 min (t(99) = 0.14, p = 0.88). This pattern did not interact (F(1, 98) = 0.12, p = 0.73) with the between-subjects factor of AI vs. human partner.

**Figure 2:**
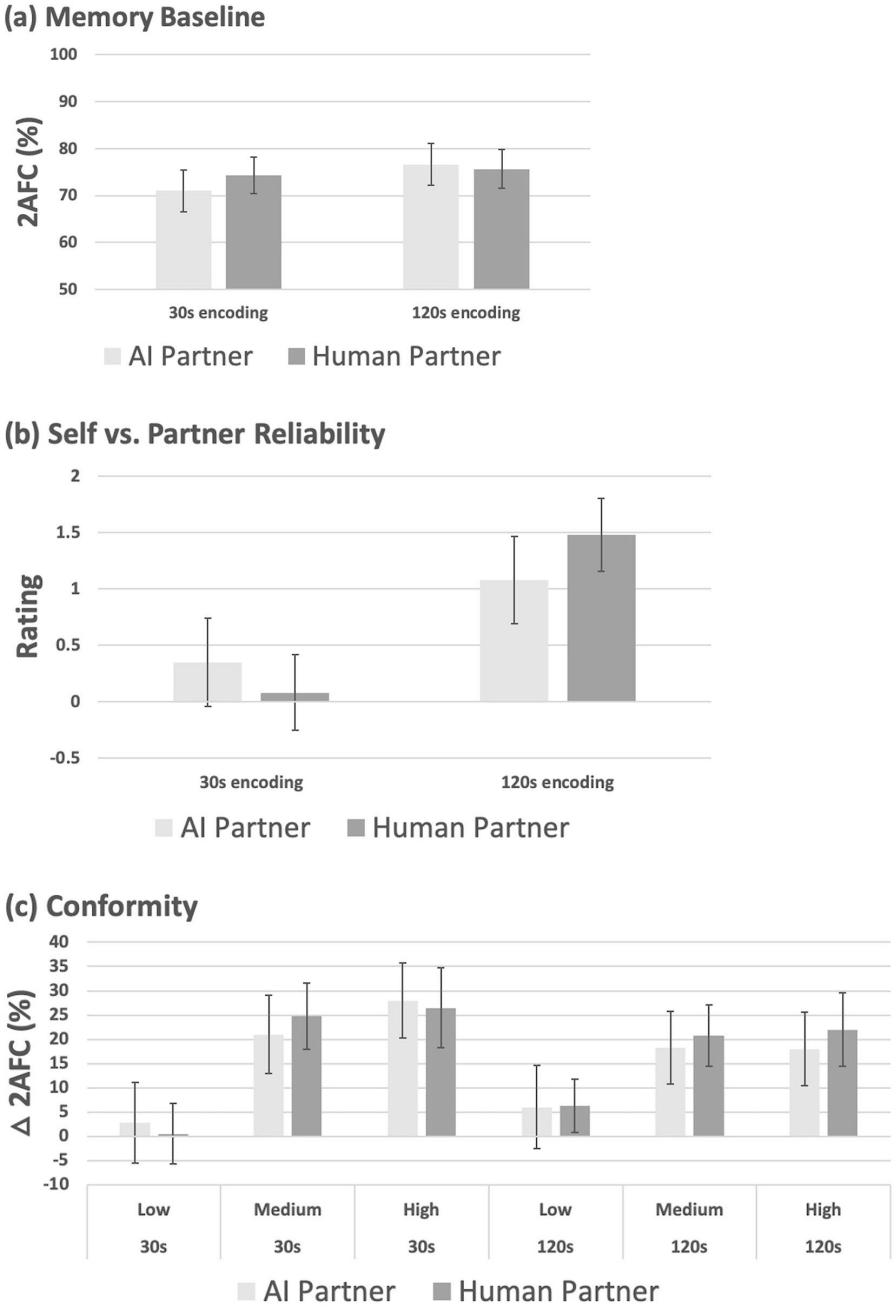
Data for Experiment 2 (Decision-likelihood effects). (**a**) Mean percentage baseline 2AFC performance (± 95% CI) following 30 s vs. 120 s viewing time at encoding, for the AI (light-grey) vs. human (dark-grey) groups. (**b**) Relative reliability ratings (± 95% CI) for participants own memory vs. AI or human partner following 30 s vs. 120 s viewing time at encoding. (**c**) Change in mean 2AFC performance (**△**2AFC, ± 95% CI) induced by conformity to accurate minus errant high, medium and low likelihood decisions from AI vs. human partner about the content of images viewed for 30 s vs. 120 s at encoding.

In Fig. 1c participants’ conformity is quantified by the spread in 2AFC accuracy after exposure to accurate minus errant decisions. Conformity is significantly increased by brief viewing times (F(1, 98) = 16.96, p = 0.00008, ηp^2^ = 0.148). This effect is marginally sensitive to the between subjects factor of AI vs. human partner (F(1, 98) = 3.09, p = 0.082, ηp^2^ = 0.031)—being somewhat more pronounced for AI compared to human decision’s (see Fig. 2c). Conformity is also significantly increased to the reputedly more vs. less accurate source (F1, 98) = 6.64, p = 0.011, ηp^2^ = 0.063), and this effect is insensitive to the between-subjects factor of partner-type (F(1, 98) = 0.017, p = 0.90). The effects of image viewing time and source reputation did not interact either with each other (F(1, 98) = 1.60, p = 0.21) or three-way with the factor of AI vs. human partner (F(1, 98) = 0.17, p = 0.68).

### Discussion

We hypothesised that AI’s informational influence is subject to a form of cognitive regulation that also constrains the influence of other humans. Supporting the hypothesis, we observed that the better-by-reputation AI and human partner had increased informational influence, attracting significantly higher levels of conformity. The identical pattern of influence suggests that similar underlying mechanisms regulate conformity to image classification decisions from AI and human sources, at least so far as we could detect here. Independently, restricted viewing time elevated conformity to decisions about briefly seen images compared to the other images. Here, we therefore find no evidence that declining capabilities lead participants to increase reliance upon sources with a better reputation. This is not due to lack of such decline—baseline was robustly lowered by 30 s vs. 120 s viewing time, and was also lower when interacting with the better-by-reputation sources. The latter may be a form of social loafing^47–49^ or automation bias^50,51^.

Restricted viewing time produced a corresponding drop in participants’ subjective rating of their memory reliability vs. partner. Moreover, when viewing time was restricted, participants subjectively rated the reputedly more accurate sources as having a better memory than those with a poorer reputation for accuracy. Evidently, this pattern of subjective effects dissociates from the pattern of informational influence as measured via conformity. Moreover, across all reputation and viewing time conditions, AI had an illusory reliability compared to human partner that also dissociates from their objective accuracy, which was identical.

In summary, knowledge about AI and human reputation for accuracy modified informational influence, exerting some measure of desirable control over participant’s conformity, but did not adjust conformity to take into account the decline in participants’ capability. Moreover, interacting with the AI generated an illusory belief about its relative reliability versus the human source that in no way reflects differences in their actual reliability which was equated and held constant—at chance overall—for the AI and human partner.

## Experiment 2: decision-likelihood effects

Experiment 1 demonstrated that conformity to AI shows a ‘classic’ pattern of functional bias towards higher credibility sources, previously observed when conforming to other humans across a wide variety of contexts over a wide variety of content^30^. In experiment 2, we now examine whether conformity is similarly biased towards specific decisions from a particular AI that are more likely to be accurate. To do this we provide information about the likelihood of each decision, to test the hypothesis that informational influence is heightened for decisions given with a higher statistical likelihood. The experiment also allows us to determine whether providing specific likelihoods allow informational influence to be further psychologically calibrated to participants’ own capabilities. That is, whether likelihoods allow for further, desirable, fine-tuning or calibration of informational influence as one’s own capabilities decline. Finally, the experiment uses a human control condition once again to test for evidence of differences in the mechanisms regulating AI vs. human informational influence.

Two tests of visual scene memory with two images in each test are employed, one scene viewed for 30 s and one for 2 min. In each test, help is provided by a single entity, either AI or human, manipulated between-subjects. Each decision has a likelihood that participants are told arises from estimates by the respective source. Three likelihood levels were used, guided by recommendations from the IPCC 5th assessment^52^ for communicating different degrees of certainty. ‘Very Likely’ decisions were estimated by the partner/AI as near 100% accurate, ‘likely’ as around 75% accurate and ‘as likely as not’ equating to 50% (chance) likelihood. Note that as in experiment 1, the objective accuracy across all decisions remains at 50%—i.e., equal numbers of accurate and errant decisions are given at each likelihood level.

### Results

Memory baseline (Fig. 2a) is reduced for briefly viewed images. This effect is significant, 1-tailed, in the predicted direction (F(1, 98) = 3.46, p = 0.066, ηp^2^ = 0.034), and does not differ for AI vs human partners (F(1, 98) = 1.33, p = 0.25). Participants’ subjective ratings of their memory reliability relative to partner (Fig. 2b) showed a robust drop for images viewed for 30 s vs. 2 min (F(1, 98) = 30.87, p < 0.00001, ηp^2^ = 0.240), an effect that appears marginally pronounced for AI vs. human (F(1, 98) = 3.05, p = 0.084, ηp^2^ = 0.030).

Conformity was strongly affected by decision-likelihood (F(2, 196) = 27.95, p < 0.00001, ηp^2^ = 0.222), being increased (Fig. 2c) to high and medium confidence decisions vs. low confidence in the 30 s and 2 min viewing time conditions. There is no main effect of viewing time (F(1, 98) = 1.05, p = 0.308), but this factor significantly interacts with decision-likelihood (F(2, 196) = 4.03, p = 0.019, ηp^2^ = 0.040). Increased conformity to decisions about the briefly vs. longer viewed images occurs in the high (t(99) = 2.19, p = 0.031) but not medium (t(99) = 1.07, p = 0.285) or low (t(99) = 1.53, p = 0.13) likelihood conditions. These conformity effects were insensitive to partner-type (maximum F = 0.322, p = 0.72).

### Discussion

As hypothesised, increasingly likely decisions had increased informational influence, but not in a strictly linear fashion. Decline in user’s own capability triggered increased informational influence for high likelihood decisions which was not observed for the medium or low likelihood decisions. Thus, the pattern of informational influence observed here, contra experiment 1, becomes sensitive to participants’ own capabilities. This further psychological calibration of informational influence reflects the same underlying mechanisms for an AI as for a human source, so far as we can detect.

Baseline 2AFC performance here was significantly reduced—in the predicted direction—by restricted viewing and, as per experiment 1, participants’ subjective ratings of their memory reliability dropped compared to the AI and human partner when images had restricted vs. longer viewing times. This was marginally more pronounced for AI, indicating some weak remnant of an illusory difference in perceived reliability of AI vs. human partner. But the previous finding from experiment 1, of AI’s illusory reliability across all conditions, is now absent.

Introducing likelihood information with each decision has therefore qualitatively changed the specific pattern of informational influence, revealing interactive control over conformity exerted by both experimentally manipulated factors as previously reported from our lab with human partners^24^. Moreover, illusory belief in the enhanced reliability of AI vs. human sources found in experiment 1 is now absent. Overall, it appears that per decision likelihood information additionally regulates informational influence, and helps bring the subjective appreciation of source reliability into alignment with the (identical) objective accuracy of the AI vs. human partner.

## Experiment 3: objective-accuracy effects

Generally speaking, a principle goal of AI research and design is to strive for accuracy appreciably higher than the chance levels employed here so far. So, it seems necessary that we conclude the present set of experiments by examining the impact that better ‘training’, leading to improved objective accuracy, has on the regulation of informational influence. To improve the objective accuracy of decisions, we simply introduced an accuracy-likelihood correlation across the low, medium and high likelihood decisions used in experiment 2. Thus, high likelihood decisions were always accurate, medium likelihood decisions were 75% accurate and low likelihood decisions were 50% accurate (i.e. actually at chance given the 2AFC memory test format).

We consider there to be two distinct predictions possible under the hypothesis that better training leads to an improved pattern of informational influence. First, conformity may become graded following restricted viewing so that it increases from low to medium to high likelihood decisions. Second, restricted viewing may induce increased use of medium and high likelihood information. A further critical question, as is the case throughout this paper, is whether differences in the pattern of informational influence will emerge for AI vs. human control.

### Results

**Figure 3:**
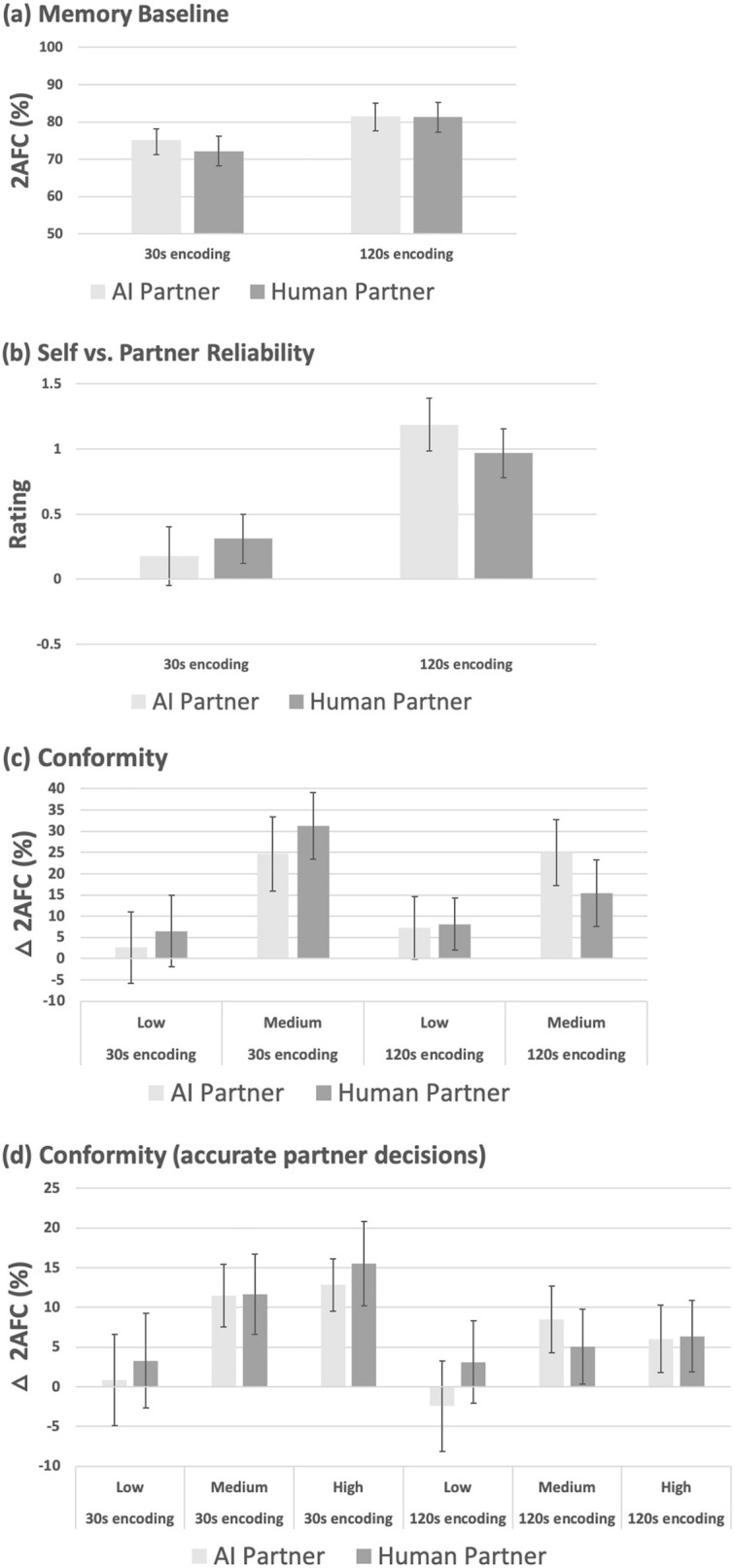
Data for Experiment 3 (Objective-accuracy effects). (**a**) Baseline 2AFC performance (± 95% CI) in the AI (light-grey) vs. human (dark-grey) groups, according to viewing time at encoding (30 s vs. 120 s). (**b**) Relative reliability ratings (± 95% CI) for participants own memory vs. AI or human partner, following 30 s vs. 120 s viewing time at encoding. (**c**)  Change in 2AFC performance (**△**2AFC, ± 95% CI) induced by conformity to accurate minus errant AI vs. human partner decisions at medium vs. low likelihood, following 30 s vs 120 s encoding of images. (**d**) Baseline-adjusted conformity-induced change in 2AFC performance (± 95% CI), for accurate AI vs. human partner decisions about image content following 30 s vs. 120 s encoding.

Restricting viewing time reduced baseline memory accuracy (Fig. 3a, F(1, 94) = 19.98, p = 0.000022, ηp^2^ = 0.175), and reduced participants ratings of their own memory reliability (Fig. 3b) compared to their AI and human partner (F(1, 94) = 21.15, p = 0.000013, ηp^2^ = 0.184). Neither effect interacted significantly with the factor of AI vs. human partner (max F = 0.955, p = 0.331).

Conformity to decisions for the medium and low likelihood conditions is shown in Fig. 3c, quantified via the spread measure calculated by subtracting 2AFC performance in accurate minus errant decision conditions as used in experiments 1 and 2. Within these data, an effect of viewing time interacts with the AI vs. human partner factor (F(1, 94) = 4.29, p = 0.041, ηp^2^ = 0.044). This main effect averages over the different likelihood levels, and it appears that viewing time overall (collapsed over likelihood) has a marginal effect on conformity to the human partner (t(47) = 1.98, p = 0.054) but not to the AI (t(47) = 0.85, p = 0.397). Likelihood information had a significant main effect (F(1, 94) = 41.58, p < 0.00001, ηp^2^ = 0.307), qualified by interaction with viewing time (F(2, 196) = 4.50, p = 0.037, ηp^2^ = 0.046) but no further interaction with AI vs. human partner factor (F(1, 94) = 1.62, p = 0.206). The likelihood by viewing time interaction reflects increased conformity to medium (t(95) = 2.06, p = 0.042) but not low likelihood decisions (t(95) = 0.97, p = 0.334) about images viewed for the brief vs. longer duration.

For accurate AI and human partner decisions the likelihood information was at a low, medium and high level (Fig. 3d), allowing us to examine whether viewing time and likelihood interact upon conformity when tested across all three likelihood levels. Both factors produced main effects (likelihood: F(2, 188) = 26.84, p < 0.00001, ηp^2^ = 0.222; viewing time: F(2, 188) = 6.14, p = 0.015, ηp^2^ = 0.061) that interacted (F(2, 188) = 4.16, p = 0.016, ηp^2^ = 0.042). High and medium likelihood decisions—but not low likelihood decisions—induced greater conformity for the brief vs. long viewing time conditions (high likelihood, t(95) = 3.71, p = 0.00034; medium likelihood, t(95) = 2.17, p = 0.032; low likelihood, t(95) = 0.68, p = 0.5). Neither the main effects nor their interaction were sensitive to the AI vs. human partner factor (maximum F = 2.22, p = 0.111).

### Discussion

As hypothesised, objectively accurate decisions with likelihoods further improved the regulation of informational influence beyond that observed in experiment 2. Here, declining memory capability resulted in increased reliance on both the medium and the high likelihood decisions, whereas in experiment 2 this was found only for the high likelihood condition. The impact of high and medium likelihood decisions on informational influence was the same for AI and human partner, providing further empirical support for the existence of similar underlying mechanisms regulating that influence from AI and from the human control. A caveat to this broad conclusion comes from the present finding that independent of decision-likelihood, the effect of viewing time on conformity was stronger for the human vs. AI source. While restricted viewing time, as in both prior experiments, caused baseline performance and the subjective reliability of memory to decline, here there is no sign whatsoever of any illusory belief in the perceived reliability of the AI vs. human partner.

As was the case moving from experiment 1 to experiment 2, here we observed further fine-tuning of conformity by the interacting experimental factors. Providing decisions with a likelihood that reflects their objective accuracy increased reliance on the objectively-accurate medium and high likelihood decisions when the participants own capabilities were weaker. Moreover, earlier signs of illusory belief in the relative reliability of AI vs. human aid were here completely absent. These findings combine to suggest that the methods in the current experiment reflect best practice out of all three, in the sense that they generated subtle but detectable improvement in psychological calibration of the source’s informational influence according to participants’ epistemic needs, and to an equivalent degree—so far as we can detect—for decisions originating with AI and the human partner.

## General discussion

From one experiment to the next, we observed an improving pattern of regulated informational influence for AI that was essentially identical to that observed with the human control. In experiment 1, where accuracy was signalled by reputation alone, AI with a better reputation were more persuasive. However, this did not increase their credibility when participants own knowledge was relatively poor, and moreover there was an overall illusory belief in the relative reliability of knowledge originating with AI vs. human control. In experiment 2, decisions came with a likelihood estimate and this allowed participants to exercise caution by conforming more only to the highest likelihood decisions when their own knowledge was relatively poor. Also, illusory belief in the relative reliability of AI transformed into a weak, marginal effect arising when participant’s own memory was relatively poor. In experiment 3, where for the first-time likelihoods objectively reflected accuracy, participants appropriately relaxed their caution and conformed more both to high and medium likelihood decisions when their own knowledge was relatively poor. Moreover, there was no sign whatsoever of any illusory belief in the relative reliability of AI.

Our primary conclusion is that informational influence from AI can be constrained and shaped in much the same way as the informational influence originating from human sources of knowledge. It’s important for us to acknowledge that this conclusion is limited by the specific interaction and content under experimental control, but the wider literature firmly suggests that this pattern of regulated informational influence is unlikely to be an artefact of the present form or content. Our AI findings, for the first time to our knowledge, conceptually replicate classic human conformity findings that are well-established and largely invariant across a variety of content in a range of natural and virtual (i.e. computer-mediated) types of interaction (reviewed in Ref.^30^). Furthermore, we also replicate and extend prior findings from our own lab^24^ showing that one’s own capability and a partner’s credibility can both interactively determine conformity.

Under this psychological regulation, informational influence becomes an increasingly fine-tuned joint function of the sources’ accuracy and a person’s own relevant capabilities. Under such control, with regard to experiment 3, it seems appropriate to conclude that the credibility of the AI source lay within its’ Goldilocks zone, producing an influence commensurate both to its likely accuracy and to the user’s epistemic need. But this was not the case in experiments 1 and 2, where the credibility of AI, and the human partner, were increased by dishonest reputational and high-likelihood estimates—that is, they were dishonest in the sense that they lacked an objective basis. Hence, experiments 1 and 2 also highlight undesirable patterns of influence that are decoupled from objective accuracy, and to that extent they are not well-regulated. It seems difficult to escape the conclusion that well-intentioned semi or partially autonomous systems should actively and intelligently strive to avoid producing such patterns of influence and, moreover, that the operational parameters of such systems should be explicitly configured with the goal to remain in a Goldilocks zone of credibility.

So far as we are aware, specific reference to informational influence as a psychological variable within human–AI interaction has been made previously only in work by Wiese et al.^53^, who investigated conformity to human and artificial agents (a computer vs. a robot hand) during an ‘analytical’ task (counting and comparing on-screen dot stimuli) vs. a ‘social’ judgement task (reading emotional states from images of eyes). While equivalent levels of conformity to all types of agent occurred on the analytical task, conformity on the social task was reduced to the least human-like artificial agent (i.e. computer vs. robot hand). Hertz and Wiese^53^ interpreted this pattern as due to the computer’s enhanced informational influence on the analytical task. But they did not include manipulations targeted at informational influence, and support for their conclusion was restricted to additional ratings data which showed that the computer’s perceived analytical capability was higher than for the robot or the human agent. Our findings here, however, appear to provide good support for the kind of role played by informational influence in increasing the credibility of artificial agents that Wiese and colleagues allude to.

Given the similar patterns of influence from human and AI sources across experiments, we conclude that similar cognitive mechanisms are likely responsible for regulating their informational influence. This proposal aligns with work demonstrating similar mechanisms at work during interactions with human vs automated agents, using neural indices derived from the electro-encephalogram (EEG) in participants monitoring an automated algorithm’s performance on a classic perceptual ‘flanker’ task’^54^. But the degree to which overlapping cognitive mechanisms support interaction with human vs. automated agents, more generally, is far from clear. Exploiting the spatial resolution of functional magnetic resonance imaging (fMRI), for example, differences have been reported^55^ in neural activity during performance of a simulated luggage-screening task where target present-absent advice comes from a human vs. machine agent. Future work is needed to specify, and even localise, the cognitive bases of interaction with human vs automated agents to gain further traction on their mechanistic basis.

In terms of mechanism, as noted in the introduction we and others have linked functional biases in conformity to explicit mentalizing mechanisms that allow us to compare our own knowledge to other people’s. Given that similar mechanisms regulate conformity to AI within the conformity paradigm, the way is now open to examine factors impacting upon AI’s operation within the Goldilocks zone of credibility. Among many, one factor that stands out is the potential impact of cognitive aging on the regulation of AI’s influence. There is growing evidence that older adults’ exhibit an altered^56,57^ or decreased^58,59,60^ ability to correctly infer trustworthy intent in others, and more widely across social media^61^, which raises the possibility of consequential age-related dysregulation of AI’s informational influence. This is particularly concerning because environmental support in older adults is important for various neural mechanisms linked to healthy aging^62^, and its provision by AI, e.g. to aid self-management in daily life or chronic disease^63,64^ may become increasingly sought after as populations age. This unfortunate constellation, comprising an increased need for systems that could produce dysregulated effects, provides a compelling case for further research using the current framework.

Our translation of social influence concepts to human–AI interaction naturally includes normative influence as an additional important player. There is a general notion of normative influence that will probably emerge as AI become more and more a part of everyday life. Which is that some measure of trust in their recommendations will become just another socially accepted way of interacting with computerised technology. But there are more specific ways in which normative influence may lead to trust in the decisions or recommendations made by AI that in our view need to be considered and investigated. From the perspective of a mechanistic understanding of how semi or partially autonomous AI influences humans, a critical issue concerns whether normative influences work through mechanisms truly independent of those that regulate informational influence. There are two broad areas of work on AI to whom this question is highly relevant. Firstly, there has been sustained interest in the use of anthropomorphic features to enhance interactions with and trust in automated technology such as robotics and now AI^65,66^. But to the extent that such features engage psychological mechanisms that mediate normative influence solely to enhance the user’s experience, they should presumably function without impact on the regulation of an AI’s informational influence. In our view, this is not a safe assumption, its’ validity and limits should be empirically established, and the current framework offers a way to do this.

Secondly, a further aspect of the relation between normative and informational influence in human–AI interaction is the recognised need^11–13^ to develop regulatory protocols and ethical-legal frameworks. From the perspective we introduce here, these may act in a manner analogous to the social norms or conventions that fuel normative influence. But, rather than effective separation between normative and informational influences as was the case above, it may instead be desirable to have effective contact between normative and informational influences to help maintain safe and legal boundaries when incorporating AI into decision-making. This means that normative guidelines and rules should function alongside, and sometimes supervene upon, the informational influence of an AI.

Take for example the medical application of image classifying AI^6–9^, or the forensic application of face recognition AI^16^—where there is currently evidence that optimal recognition involves collaboration between AI and human experts^67^. In each context, informational influence may be formed as decisions provided by AI combine with practitioners’ expert knowledge in cases/crimes varying in severity or risk. Professional protocols in each context may have a common aim, to modify the response to identifications with a given likelihood according to the severity of the case/crime under investigation. For example, when assessing life threatening disease outcomes, the threshold for action might be lowered (i.e. decisions with lower likelihood might (normatively, according to a protocol) be deemed acceptable. This is a form of normative influence, driving adherence to a convention for use intended (in this example) to supervene upon informational influence. The current framework provides the conceptual and empirical tools to study how such normative and informational influences interact, in many different contexts, so that lessons learned in one can help to inform others.

To conclude, we began this paper citing the rising optimism that coexists with concerns about AI, and the consequent need to understand how we may reap the benefits and avoid or at least minimise the costs. A recognised^11–13^ part of the solution is regulatory ethical and legal frameworks, enacting fair and widely acceptable principles such as safety, transparency and responsibility in alignment with positive human values, as articulated for example in the 23 Asilomar principles^68^. Our contribution here is to propose what is in effect a regulatory principle for the ethical and legal operation of semi or partially autonomous AI. Namely, that their use remains within a Goldilocks zone of credibility—where a systems’ power to persuade is desirably yoked to its accuracy in conjunction with the shifting epistemic need of human users. This unavoidably brings human psychology into the equation where, fortuitously, there already exist functional mechanisms that evolved to regulate human agents’ informational and normative influence. Our experimental evidence strongly suggests that similar functional mechanisms regulate the influence of AI agents providing image recognition and classification decisions to support memory-based judgements, and we believe that this approach can and should now be used more widely to investigate when an AI is operating within a Goldilocks zone and when it is not.

It seems inevitable, perhaps, that AI will have an increasing societal-level impact, and for that reason it is in our view important to pay attention to whether or not semi and partially autonomous AI operate within a Goldilocks zone of credibility. In particular, this should help to identify where AI inadvertently create or exacerbate gaps in transparency, safety and ethical standards. Robust indicators of operation inside, or outside, the goldilocks zone of credibility could therefore in due course contribute metrics that help to assess the societal-level impact of AI. A notable example being the impact of AI on progress towards sustainable development goals^1,69^ that entail the minimisation or elimination of such gaps.

## Methods

### Ethics statement

All experimental protocols were approved by the University of Aberdeen School of Psychology Ethics Committee, in accordance with the British Psychological Society (BPS) Code of Human Research Ethics, and carried out in accordance with University of Aberdeen research governance procedures. In accordance with the local ethics committee procedures and the BPS Code of Human Research Ethics, we obtained informed consent from all participants, all of whom were aged 16 or over.

### Participants

Across all three studies a total of 296 volunteers participated (224 female, 72 male), aged between 16 and 39 years (experiment 1 mean age 20.3 years (SD = 2.78), experiment 2 mean age 20.5 years (SD = 2.30), experiment 3 mean age 21.3 years (SD = 3.18)). Within each experiment participants were randomly assigned either to the AI or Human partner conditions: experiment 1, AI N = 52, human N = 48; experiment 2, AI N = 50, human N = 50, experiment 3, AI N = 48, human N = 48).

### Stimuli and apparatus

Each experiment used a set of six different hi-resolution bitmap images showing different household scenes^24,34^ including a kitchen, a bedroom, a bathroom, an open wardrobe, an office and an open toolbox. Each participant viewed two different scenes picked at random from the image pool in each of the two encoding-retrieval blocks described below. In each block, memory for scene details was tested using thirty 2-alternative forced choice (2AFC) questions per scene. To illustrate, participants were cued with: “Bathroom-Window: Open/Closed; or “Kitchen-Kettle: Black/Gray; or “Bedroom-Fruit: Apple/Banana”.

Each experiment was implemented by modifying experimental scripts employed in earlier memory conformity studies^23^ written in the EPRIME v2.0 E-Studio package. The chief modification was to include instructions specifying the characteristics of the AI vs. human partner. Data collection sessions were run with groups of participants in different computing laboratories, equipped with multiple desktop PCs and identical monitors, each running the experimental scripts within the terms of E-Prime’s network licence.

### Trial types

Four trial types were used in each experiment. “Instruction” trials for each experiment are described in the experiment specific protocol section below. On “Encoding” trials, a specific image was viewed for one-of-two specified durations (either 30 s or 2 min) in each experiment. Each encoding trial began with a blank screen for 3 s, and then the visual image centred at the screen centre for a software controlled duration of either 30 s or 2 min. No overt response was required. An inter-trial interval of 5 s with a blank screen was interposed between each encoding trial. On “Retrieval” trials, a 2-alternative forced choice (2AFC) cued recall memory task was administered. On each retrieval trial the name of the image was shown centred at the top of the screen. Underneath that a cue for specific image details was shown, and underneath that separated to the left and right of the screen midline the two alternative response options were given, one correct and one incorrect (all text in uppercase Arial font). This cueing information was shown for 3 s, and then the simulated partner/AI decision was indicated onscreen by underlining one of the response options. The positioning of the correct option to the left or right was controlled by counterbalancing. Across experiments, the proportion of correct, incorrect and not-shown (i.e. baseline trial) decisions, as well as presentation of associated likelihood information, varied as described below in the specific experiment protocol section. Responses were made via keyboard, to select either the left (using the “Q” key) or the right (using the “P” key) hand option. No time limit for responding was imposed.

The 4th remaining trial-type measured subjective “Memory Reliability” for each specific image via a 7-point Likert-scale. Participants rated the reliability of their own memory per image compared to that of their partner. The name of each image was shown at centre screen, and underneath that the Likert response scale was shown, anchored on the left with “Self” and on the right with either “AI” or “Partner”. The 7-point scale ran from Self—much more reliable, somewhat more reliable, a bit more reliable), to AI/Partner—a bit more reliable, somewhat more reliable, much more reliable) with “equivalent” as the mid-point response option. Responses were given by mouse click on the appropriate response text. No time limit for responding was imposed.

### General task protocol

Each experiment consisted of two blocks, and each block comprised of general orienting instructions, specific encoding instructions followed by two encoding trials. This was followed by retrieval instructions, and then 60 retrieval trials. At the end of each block two relative reliability trials were given, one for each encoded image. A 1 min rest was then given, and then the second block—with an identical structure—began. In total each group session took around 40 min, including circa 5 min at beginning and end for allocating participants to PCs and final debriefing.

On arrival, participants were initially instructed that the purpose of the experiment was to examine how artificial intelligence/another human aids our decision-making. In the AI Sessions, it was further explained that the AI device had been trained to perform image interpretation. It was then explained that the experiment contained two blocks in each of which two images would be shown for different lengths of time. The two encoding trials were then given. Instructions specifying the capabilities of the AI/human partner, and the retrieval instructions were then given following a minute-long break after the encoding phase was complete. These instructions varied in each experiment, as explained in the specific experiment section below.

Prior to each retrieval phase, a practice retrieval task was given consisting of five simple general knowledge questions with 2-alternative forced choice response options. No responses from the partner were given to these practice questions. Within the following 60 retrieval trials, 30 were for items belonging to each of the two just encoded images, and image-order was randomised. On the following two relative reliability trials, the self/partner relative reliability likert response was administered for each encoded image in turn, in a randomised order. A 1 min rest break was then provided, before the 2nd identically structured block was given.

Finally, participants were thanked for their participation and fully debriefed as to the purpose of the experiment using a standardised form approved by the Aberdeen School of Psychology ethics committee. During debrief, participants were given the opportunity to freely express their views about the experiment so that we could determine whether there were any issues with not following instructions, or scepticism about the origin of the information received from a human partner or from AI, but no such issues emerged.

### Experiment-specific protocols

Each experiment adhered to the general protocol above, with two exceptions. First, the accuracy/decision likelihood characteristics of the AI/human partner were conveyed in different ways in experiment 1 vs. experiment 2 and 3. Second, in experiment 2 there was no correlation between objective accuracy and the levels of likelihood given with each decision, whereas in experiment 3 the two were correlated. Both of these exceptions are explained in turn in detail below.

In experiment 1, accuracy characteristics of the human partner/AI were described solely by a reputational label, indicating either accuracy on average in ‘6 out of 10 cases’ vs. ‘8 out of 10 cases’. This information was provided after the Encoding trials and immediately prior to the retrieval trials in each block. The allocation of 6 vs. 8 out of 10 labelling was counterbalanced so that half the participants received 6 out of 10 prior to the memory test in the first block, and half prior to the memory test in the 2nd block. No further accuracy or likelihood information was provided with each decision, and the overall objective accuracy of the partner/AI decisions was 50%. That is, for each encoded image, the AI/partner provided accurate answers to 10 of the 30 questions, incorrect answers to a further 10 and on the remaining 10 no answer was given. The mapping of these three decision conditions (none (baseline), accurate, errant) to each specific 2AFC question was randomised for each image.

In experiment 2 the overall accuracy labelling from experiment 1 was removed entirely. Instead, each of the 60 decisions on each retrieval trial in both blocks was conveyed with likelihood information using guidelines for imparting degrees of certainty based on statistical evidence provided in the IPCC 5th assessment^52^. Participants were told that each of the partner’s responses would be conveyed with a likelihood estimated by the AI/Human. Participants were instructed that decisions labelled as being ‘very likely’ conveyed information whose accuracy the partner/AI estimated to be near 100% accurate. The ‘likely’ label conveyed information estimated at around 75% accurate, and the ‘as likely as not’ label was for decisions about which estimated accuracy was chance—i.e. 50%. The overall objective accuracy of the partner/AI information remained at 50% as per experiment 1, because the proportion of accurate, incorrect and no-responses remained the same and was randomised across items for each image as per experiment 1.

In experiment 3, all details were as per experiment 2, except here the number of correct and incorrect responses covaried with the likelihood information and in order to achieve the necessary proportions the associated trial numbers belonging to accurate/inaccurate/baseline trials was changed as follows. Per image, responses on eight trials were labelled as being ‘very likely’ and all of these responses were accurate. Per image, a further eight responses were labelled as being ‘likely’ and of these 6 (75%) conveyed accurate information and 2 (25%) did not. Per image, a further 8 responses were labelled as being ‘as likely as not’ and of these 4 (50%) conveyed accurate information and 4 (50%) did not. This left a remaining 6 (out of 30) trials per image, to estimate baseline performance when no partner/AI response is given.

### Data analysis

All measures were derived after each data collection session was complete from the EPRIME data file created for each participant during the session. These were first written to a network drive and then merged using EPRIME’s Emerge utility. The merged data files were analysed to derive the performance measures using a standard set of bespoke analysis scripts within EPRIME’s EDAT utility. SPSS version 26 was used to perform all statistical analyses. Memory on the 2-alternative cued recall task was quantified by mean accurate performance per encoded image for each subject in each of the AI/human response conditions employed in each experiment. Relative reliability ratings for each image were quantified by assigning a score of 7 to Self-much more reliable, and descending by 1 for each subsequent likert response option. For analysis, 3.5 (the mid-point score indicating equivalent reliability of Self/Partner memory) was firstly subtracted in order to zero the scores at the point of equivalent reliability. Hence, scores above zero indicate relatively higher perceived reliability of Self vs. Partner, and scores below zero indicate relatively higher perceived reliability of Partner vs. Self.

### Statistical analysis

Our focus is on quantifiable change in the informational influence of human vs. AI in each experiment, as measured by induced changes from baseline performance when partners’ decisions are provided before the participants give their own response. These induced changes elevate or reduce performance relative to baseline when the partner response is accurate or incorrect, respectively. This produces a ‘spread’ around that baseline. We used this spread measure in each experiment to quantify informational influence. The key analyses in each experiment concern the effect of viewing time at encoding and the effect of the accuracy labelling (experiment 1) or likelihood information (experiments 2 and 3) upon the spread induced by the AI vs. the human partner. To capture these effects we used a mixed design ANOVA, with partner type (AI vs. Human) as a between-subjects effect and, within-subjects, repeated measures effects of viewing time at encoding (30 s vs 2 min), credibility (6 vs. 8 out of 10—in experiment 1) or decision-likelihood (low, medium, high—in experiments 2 and 3). We report all the main effects and interactions involving these factors including their effect sizes via the partial eta-squared statistic (ηp^2^). Conventionally significant (p < 0.05) results were followed up by specific planned t-tests.

## Data Availability

The data are available from the corresponding author on reasonable request.
